# The Effects of Airflow on the Mechanosensitive Channels of *Escherichia coli* MG1655 and the Impact of Survival Mechanisms Triggered

**DOI:** 10.3390/microorganisms11092236

**Published:** 2023-09-05

**Authors:** Violette I. Ramirez, Robin Wray, Paul Blount, Maria D. King

**Affiliations:** 1 Department of Biological and Agricultural Engineering, Texas A&M University, College Station, TX 77845, USA; 2 Department of Physiology, University of Texas Southwestern Medical Center, Dallas, TX 75390, USA

**Keywords:** airflow stressors, AMR genes, bioaerosol resuspension, mechanosensitive channels

## Abstract

**Highlights:**

What are the main findings?

- Aerosolization and ventilation air velocity affect antibiotic resistance of *E. coli* strains;
- Bacteria respond to ventilated environments through mechanosensitive ion channels triggered by aerosolization;

What is the implication of the main finding?

- Critical infrastructures require real-time knowledge about their environment for microbiome source tracking;
- Clinical indoor spaces where bacterial infections are treated can result in potential exposure to bioaerosols.

**Abstract:**

Understanding how bacteria respond to ventilated environments is a crucial concept, especially when considering accurate airflow modeling and detection limits. To properly design facilities for aseptic conditions, we must minimize the parameters for pathogenic bacteria to thrive. Identifying how pathogenic bacteria continue to survive, particularly due to their multi-drug resistance characteristics, is necessary for designing sterile environments and minimizing pathogen exposure. A conserved characteristic among bacterial organisms is their ability to maintain intracellular homeostasis for survival and growth in hostile environments. Mechanosensitive (MS) channels are one of the characteristics that guide this phenomenon. Interestingly, during extreme stress, bacteria will forgo favorable homeostasis to execute fast-acting survival strategies. Physiological sensors, such as MS channels, that trigger this survival mechanism are not clearly understood, leaving a gap in how bacteria translate physical stress to an intracellular response. In this paper, we study the role of mechanosensitive ion channels that are potentially triggered by aerosolization. We hypothesize that change in antimicrobial uptake is affected by aerosolization stress. Bacteria regulate their defense mechanisms against antimicrobials, which leads to varying susceptibility. Based on this information we hypothesize that aerosolization stress affects the antimicrobial resistance defense mechanisms of *Escherichia coli* (*E. coli*). We analyzed the culturability of knockout *E. coli* strains with different numbers of mechanosensitive channels and compared antibiotic susceptibility under stressed and unstressed airflow conditions. As a result of this study, we can identify how the defensive mechanisms of resistant bacteria are triggered for their survival in built environments. By changing ventilation airflow velocity and observing the change in antibiotic responses, we show how pathogenic bacteria respond to ventilated environments via mechanosensitive ion channels.

## 1. Introduction

Antimicrobials, including antibiotics, are medicines administered to treat bacterial infections in humans, animals, and plants. The misuse of antimicrobial drugs, like antibiotics, has introduced the rise of antimicrobial resistance in bacteria and thus created unexpected complications [1]. This has led to microorganisms reacting unresponsively to drug treatments, leading to infections, spreading illnesses, and in some cases, leading to death. It has been reported that over 100,000 people die every year in US hospitals alone due to untreatable bacterial infections [2]. Microbial exposure varies depending on seasonal variations influenced by temperature, relative humidity, and air exchange rates [3]. Medical facilities have reported community-related methicillin-resistant *S. aureus* (CA-MRSA) pathogens transmitted via air movement exposure [4]. The ability of airborne pathogens to be transported due to air movement has raised questions about the accurate enumeration of microorganisms present. Studying bioaerosols around field sites is particularly useful for monitoring environmental factors, as well as for detecting microbial sources and movement [5]. Based on previous studies, researchers concluded that there is evidence of an association between bioaerosol levels and environmental factors, such as temperature and relative humidity [2]. Furthermore, there is evidence that some causes of infections include mechanical ventilation and time [6,7].

Along with understanding bioaerosol levels comes quantifying and characterizing various microbes. Bioaerosols can be categorized into two main groups: viable and nonviable. Viable organisms maintain culturability, metabolic activity, and membrane integrity, whereas nonviable organisms do not. Additionally, viable bioaerosols can be classified as either culturable (VBC) or nonculturable (VBNC). Specifically, culturable bioaerosols encompass fungi, viruses, and bacteria microorganisms that can reproduce in controlled environments [8]. However, microbial plating cannot detect viable but nonculturable (VBNC) bacteria [9]. It is important to recognize that stressed bacteria are harder to enumerate as they are undetectable by current molecular methods. According to a study of airborne microbial communities, airborne microbial community composition and dynamics continue to be inadequately described [10,11]. This is because cultivable microbes constitute only a small fraction of the total airborne microbes present [12]. Airborne bacteria undergo large stresses from the absence of ideal conditions, warm temperatures, and hydration. Notably, bacteria can develop resistance to certain stresses due to stress response genes [2]. Stress from aerosol generation can lead to genome rearrangements or support mutations that increase antibiotic resistance [2].

Understanding how the survival defense mechanisms of bacteria are triggered is of crucial importance for preventing new generations of bacteria with antimicrobial resistance (AMR). Our research focuses on how bacteria react to environmental conditions and defend themselves. The goal of this research is to characterize AMR and its mechanisms as a response to sonic airflow velocity at 313 m/s, near the speed of sound. Information about aerobiology mechanics influences the conditions of sterile ventilated environments. Rapid air flow movements in ventilated areas are of interest because of the high stress that microorganisms are subjected to and their ability to survive and duplicate.

Trying to balance our interactions with the microbiome we live with has historically been rooted in ensuring the safety of public health. Identifying the survival mechanisms of bacteria and how they develop resistance to current safety measures and environmental conditions, including high ventilation airflow, continues to be an important question. From single-celled bacteria to multicellular animals and plants, they all respond to mechanical forces in their extrinsic environments and internal environments for true development and health [13]. Similarly, proteins and protein complexes can respond to these mechanical forces. MS channels expose this phenomenon by operating between membrane mechanical properties and protein structure and function. These channels were first documented on the surface of *Escherichia coli* (*E. coli*) spheroplasts via the patch-clamp technique, a method that requires a fine glass pipette to be in close contact with the cell membrane using a microscope and micromanipulator [14]. Electrophysiologically, four activities are observed in *E. coli* native membranes: the largest conductance channel of about 3.6 nanosiemens, MscL; two activities about a third of that conductance, MscS (from a gene previously called yggB) and MscK (previously kefA), with the only distinction being that MscS more readily inactivates; and a fourth activity about a third of the conductance of MscS/MscK, MscM [15,16]. While MscL is an independent family of channels, MscS, MscK, and MscM are all related, and there are three additional paralogues in *E. coli* that encode channels only observed by patch clamp when overexpressed [17].

It is important to recognize membranes as dynamic mediums that explicitly influence the function and spatial distribution of the surrounding proteins [18]. This relationship is exemplified by the two-channel proteins found in the plasma membrane of *E. coli*: the mechanosensitive channels of large conductance (MscL) and small conductance (MscS). MscL channels serve as an osmotic emergency release valve that stops cell lysis upon acute decreases in the osmotic environment. According to previous studies, MscL and MscS directly respond to differences in membrane tensions by opening nanoscale protein pores [19,20]. Since the discovery of MS channels, many studies have investigated the genes and crystal formation of open and closed pore structures. MscL has the biggest pore size of the gated ion channels and is approximately 28 Å when fully opened [21,22,23]. As of today, bacterial MS channels are the only bacterial channels with a defined physiological function [20]. It is important to mention that MscL channels are the only known microbial channels where an activity, protein structure, protein dynamics, and physiological role with homologues have all been incorporated [20,24].

Despite MscL being a unique homologue, it has the structural/functional essence that occurs in higher organisms and can clarify how more complex channels function. Researchers have highlighted the features of MscL which include (1) the potential to sense and react to biophysical changes in the membrane, (2) an ⍺ helix or sequence of charges at the cytoplasmic membrane border to assist transmembrane development, and (3) principal subunit interfaces that determine if the channel gates open appropriately when they interact [20]. Bacterial channels present a target of interest for drug delivery methods, especially because of their high measure of conservation in bacterial species and absence from the human genome [24,25]. Since these MS channels have evolved to detect instinctive tension in the membrane and transmute it into an electrochemical response, they can act as arrival mechanisms for drugs and other small molecules into bacterial cells [13].

However, the physiological functions of many other channels of bacterial species remain a mystery; linking channel genes with the physiology phenomena is yet to be answered. The answer may lie within the bacterial mechanosensitive (MS) channels that can sense tension in the membrane and act as emergency release valves, allowing solutes to quickly leave the cytoplasm and dissipate osmotic disparity between internal and external environments [26]. Studies have already identified structural genes encoding various MS channels, but their physiological role is still unclear [15]. In this study, the exposure of aerosolization to *Escherichia coli* in a controlled ventilated environment will clarify if mechanosensitive ion channels play a role in the physiological response to antimicrobials. By observing the antimicrobial response of stressed microorganisms, we can observe if the MS channels present in *E. coli* influence survival mechanisms leading to antibiotic resistance.

Mechanosensitive (MS) channels in *Escherichia coli* provide protection against hypoosmotic shock [15,20,27]. These response mechanisms have evolved with bacteria because of the extreme environmental conditions they live in. Channels and sensors are likely to have common gene ancestors and therefore share the same features [20]. Proteins are in fixed locations in membranes and are subjected to anisotropic forces by their environment. Mechanosensors can sense changes in the membrane tension and are the guide for basic biological functions. Recent publications are focused on understanding the molecular mechanisms of mechanosensation. The mechanosensitive channels of large and small conductance were found in closed, open, and adaptive states [15,28,29]. Often studies reported several structures of MscS in closed or open confirmations, but it is unclear how sample conditions in these studies determined certain conformational changes [30]. As of today, it has been demonstrated that reversible lipid removal and addition determine whether MscS is an open or closed conformation [30]. Genes have the material required to create functional proteins and studies suggest that the degree of dilapidation of a membrane protein depends on the exact conditions of lipids [30]. MscL is the largest gated pore and can pass 30 Å molecules. These channels have evoked interest because of their ability to directly sense and respond to bio-physical changes in the membrane and protein structure [20]. Bacteria can protect itself to a varying degree via gating ion channels when subjected to mechanical stress. The shear stresses cause physical impairments to the bacterium cell wall [31,32] and trigger a reaction response. Based on this information, we test if the absence of the genes responsible for making the MscS/L protein affects the antimicrobial response of *E. coli.*

Our preliminary results suggest that aerosolization triggers intrinsic defensive mechanisms in bacteria to maintain homeostasis to survive stressed environments. Based on this hypothesis, we determine if the mechanosensitive channels of *E. coli* strains influence antimicrobial resistance with increased sonic air velocity. The goal of this study is to delineate the defensive mechanisms of different aerosolized bacteria strains and their relationship to antimicrobial resistance (AMR) by quantifying and comparing the AMR response of *E. coli* knockout mechanosensitive strains and identifying their role in changes with AMR susceptibility.

## 2. Materials and Methods

### 2.1. Bacterial Strains and Growth Conditions

The bacterial strains used in this study include *Escherichia coli* MG1655, Frag1, MJF367, MJF451, MJF465, MJF641, and MJF455, environmental isolates with knockout mutant genes [17] (Table 1). *E. coli* strains were selected for the study due to the sensitivity of the parent strain to the antibiotics used in the study and the availability of the channel knockout strains that were prepared by the protocols described in previous studies [15]. All strains were grown at 37 °C in Luria-Bertani (LB) broth. Real-time polymerase chain reaction (qPCR) of *E. coli* was performed to confirm knockout gene strains. Controlled environmental conditions include airflow, temperature (22 °C), relative humidity (80%), and light; these conditions were kept by completing tests inside a biosafety level 2 cabinet with monitored sensors. All *E. coli* strains were subjected to mechanical and sonic stress at 313 m/s velocity facilitated by the SKC Bio-Sampler Impinger at these conditions.

### 2.2. Sonic Air Velocity—SKC Bio-Sampler

The sonic air velocity was created using the SKC Bio-Sampler at the volumetric flow rate of 12.5 L/min, with each of the critical (sonic) orifice nozzles permitting 4.2 L/min of ambient air to pass through, with the diameters (3) specified as 1.27 mm. The near sonic air velocity in the nozzle openings of the bio-sampler is 313 m/s [33]. At this speed, due to the high shear forces acting on the membrane of *E. coli*, cell integrity could be compromised and enter an unfavorable state.

### 2.3. Site Description—Model Chamber

**Figure 1:**
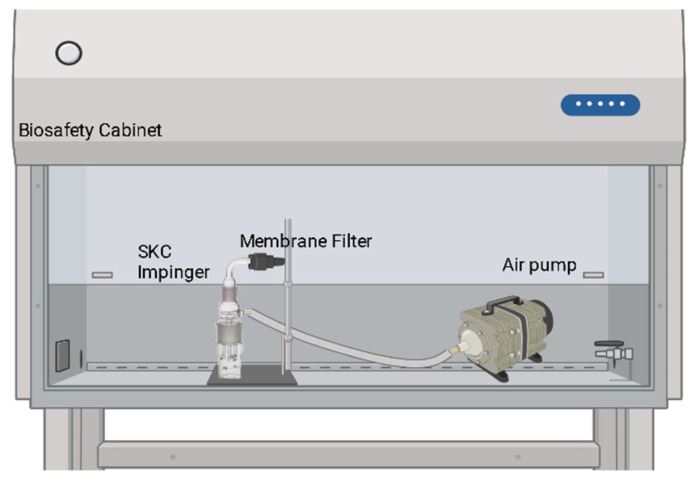
Schematic of the experimental setup in a biosafety cabinet for *E. coli* MG1655, MJF429, and MJF465 SKC Bio-Sampler Impinger.

The sonic stress experiments were performed with the SKC Bio-Sampler impinger connected to an air pump placed in a biosafety level 2 (BSL 2) cabinet while aerosolizing *E. coli* MG1655, Frag1, MJF367, MJF451, MJF465, MJF641, and MJF455 strains at 0 (control), 5, 10, 20 and 30 min (Figure 1). During testing, the temperature in the cabinet was maintained at 25 °C, at a relative humidity of 60%, with the light on and the blower constantly operating.

### 2.4. Aerosolization and Collection

The aerosolization tests were completed by using an air pump at 12.5 L/min connected to the SKC impinger full of 20 mL of fresh, mid-log phase *E. coli* MG1655, Frag1, MJF367, MJF451, MJF465, MJF641, and MJF455 pelleted at 2880 g for 7 min and resuspended in 30 mL of 10% Phosphate Buffer Saline (PBS) buffer with pH 7.4 at room temperature (RT, 25 °C). Determined by the test time, the air pump was turned on for 5, 10, 20, or 30 min. Of the fresh batch stock suspension at OD 0.7 (~10^9^ mL concentration), 150 mL was used for each test. Of the 150 mL stock, 20 mL aliquots were used for each stress test at 0, 5, 10, 20, and 30 min. The weight of the initial 150 mL stock was measured as well as the initial weight of each 20 mL batch before and after the sonic stress testing to verify minimal loss. Of the 20 mL suspension, after each test, 100 μL of serial dilutions of 1 × 10^−4^ and 1 × 10^−5^ were used for plating; these two dilutions were used for counting the colony-forming units.

### 2.5. Plating and Analysis

After the collection and dilution of bioaerosol stressed and unstressed samples, the *E. coli* samples of each stressed and unstressed strain were gravimetrically measured and plated in appropriate dilutions on Tryptone Soy Agar (TSA) plates in three replicates per media. To minimize osmotic stress, the TSA medium was selected due to its lower NaCl concentration (5 g/L) compared to the 10 g/L in the commonly used Luria-Bertani medium. After overnight incubation at 37 °C, the colony-forming units (CFU) were counted on the plates. The cultures for antibiotic resistance testing were started in Luria-Bertani medium for each *E. coli* strain, and the aerosolization time was incubated overnight at 37 °C to be spread on TSA with specific antibiotics for the colony forming units (CFU). CFU were counted on five commonly used antibiotics: 30 µg of Tetracycline (TE-30), 10 µg of Ampicillin (AMP-10), 10 µg of Gentamicin (GM-10), 50 µg of Kanamycin (KAN-50), and 25 µg of Chloramphenicol (CHL-25 or Chlora-25).

Statistical significance was designed to evaluate the expressed resistance differences in the stock samples and collected aerosol samples. ANOVA was used to determine statistical significance. The mean resistance expression of stock samples equals the mean resistance expression of the collected aerosol samples is the null hypothesis.

### 2.6. DNA Extraction

DNA extraction was accomplished by initially pelleting cells at 13,000 g from 1.5 mL aliquots for each sample, followed by subsequent cell lysis, protein denaturation, and DNA purification [34]. Using sterile TENS buffer containing Tris-HCL, Ethylenediaminetetraacetic acid (EDTA), NaOH, and Sodium Dodecyl Sulfate (SDS) (VWR, Radnor, PA, USA), pelleted cells were lysed for 10 min. For protein precipitation, a 1 M filter of sterilized sodium acetate was added and the supernatant holding the DNA was moved to a sterile Eppendorf tube. To precipitate the DNA, ice-cold isopropanol was used for an hour on ice, followed by centrifugation at 13,000× *g*. Ice-cold 70% ethanol was used to wash the pellet. After the final pellets were dried, the purified DNA was resuspended in 25 µL of 0.22 µm filter-sterilized deionized water. For each sample, DNA extraction was completed in three replicate samples and quantitated using a Nanodrop UV-Vis spectrophotometer (ThermoFisher Scientific, Waltham, MA, USA).

After isolation and purification, the replicates and time points for each aerosol sample of DNA were used for amplification in the quantitative Polymerase Chain Reaction (qPCR) to validate the presence or absence of MS channels.

### 2.7. Quantitative Polymerase Chain Reaction (qPCR)

To confirm the molecular signatures of mechanosensitive genes responsible for coding the mechanosensitive proteins, the target genes, mscL, mscS, and mscK, were amplified and analyzed in the *E. coli* strains MG1655, Frag1, MJF367, MJF451, MJF455, MJF465, and MJF641. The PCR result combination (25 μL total) included the DNA template (5 μL), the forward and reverse primers (1 µM, 1 μL each), 100 mM deoxyribonucleotide triphosphate (dNTP) mix (1 μL, Promega Corp., Madison, WI, USA), 10× PCR buffer (2.5 μL, Promega), 13.5 μL filter-sterilized water and Taq polymerase (1 μL, Promega). To measure the mechanosensitive channel of small resistance protein gene, the *mscS* forward ‘5-TAT CGC GCG GAT GAT TTC CA’ and reverse ‘5-GGT TAG ACA GTG ACC CCT GC’ primers (Integrated DNA Technologies, Coralville, IA, USA, IDTDNA) were used to amplify a 224 bp fragment [35]. To test the mechanosensitive channel of the large resistance protein gene, the mscL forward ‘5-GTC TCT TCA CTG GTT CCG A’ and reverse ‘5-TGC ATC ACA GCA GGG AT’ primers (Integrated DNA Technologies, IDTDNA) were used to amplify a 125 bp fragment. Amplification using the ABI GeneAmp 9700 thermal cycler (Applied Biosystems, Life Technologies, Waltham, MA, USA) allowed for 5 min of denaturation at 94 °C, 30 cycles of annealing at 94 °C for 30 s, 53 °C for 30 s, and 72 °C for 120 s, followed by an extension of 10 min at 72 °C, and a final holding temperature of 4 °C. The occurrence of the target DNA amplicons was then examined using agarose gel electrophoresis.

The quantitative PCR (qPCR) reaction mixture (10 µL total) contained the DNA template (3 µL), the forward and reverse primers (1 µL each), and Power SYBR Green PCR 2 × Master Mix (5 µL, Life Technologies, Waltham, MA, USA). Amplification and quantitation consisted of 10 min of denaturation at 95 °C, 40 cycles of annealing at 95 °C for 15 s, 60 °C for 60 s, and finally a holding temperature of 4 °C.

## 3. Results

### 3.1. Environmental Conditions

The sonic pressure aerosolization of *E. coli* strains by the air pump facilitated the environmental stressors while the bio-safety cabinet maintained constant temperature and relative humidity.

### 3.2. Culturability

The stock culture of *E. coli* MG1655, Frag1, MJF367, MJF451, MJF465, MJF641, and MJF455 at 0 min were used as controls for unstressed conditions. The culturability of the stock controls without aerosolization was used as the control CFU/mL as a reference for statistical analysis.

### 3.3. Culturability of E. coli Strains

**Figure 2:**
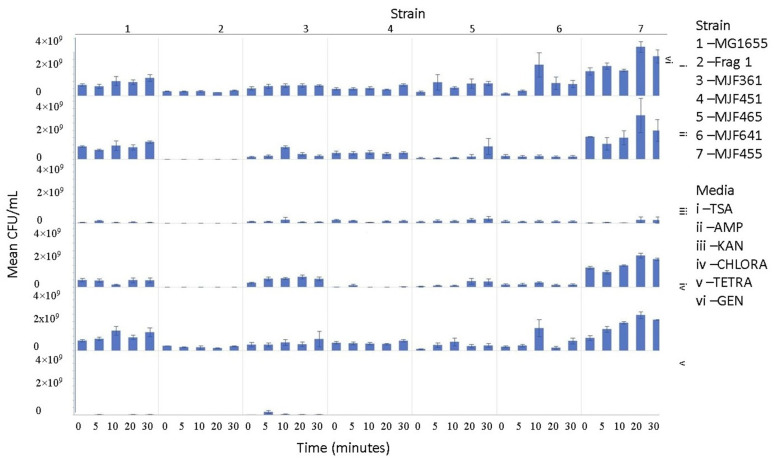
The mean CFU/mL values (left *Y*-axis) are shown at 0, 5, 10, 20, and 30 min (bottom *x*-axis) for strains 1, 2, 3, 4, 5, 6, and 7 (top *X*-axis) and media i, ii, iii, iv, v, and vi (right *Y*-axis).

The culturability and colony-forming units (CFU/mL) of each nebulized *E. coli* strain for different periods of time were examined. The culturability of the aerosolized *E. coli* strains was measured after exposure to sonic air velocity for different periods (0 min–30 min). The culturability trend, based on ANOVA hypothesis testing (between strains), varied significantly depending on the *E. coli* strain selected (Figure 2).

**Figure 3:**
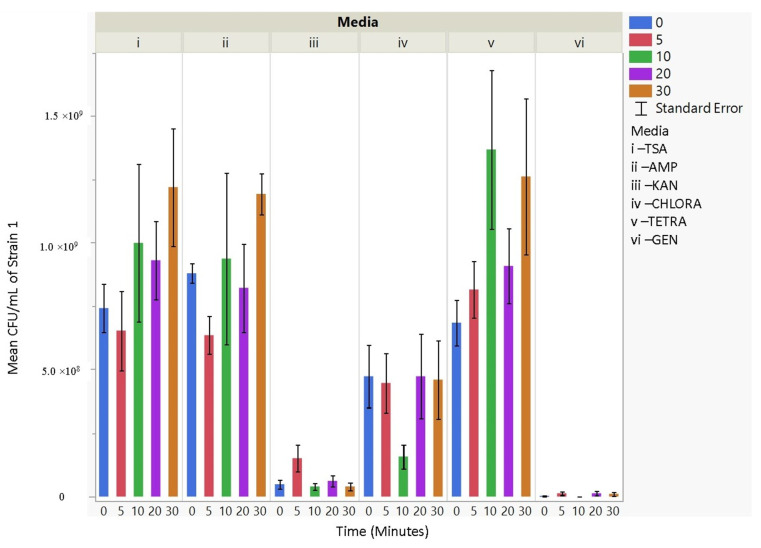
The mean CFU/mL values (left *Y*-axis) are shown for strain 1, MG1655, at 0, 5, 10, 20, and 30 min (bottom *X*-axis) per media (i, ii, iii, iv, v, and vi) (top *X*-axis).

Upon aerosolization stress, the Frag1 parent strain shows tetracycline sensitivity, as does the MscS null, but the latter also shows an increased sensitivity to kanamycin (Figure 3). However, when combined with other nulls, particularly MscL or MscM, tetracycline resistance is observed, not sensitivity. This suggests that the absence of these channels reverses the effects of tetracycline. In addition, the kanamycin sensitivity that is observed for MscS null is lost when combined with MscL or MscM nulls, further implicating complicated physiological interactions. Finally, ampicillin and chloramphenicol resistance are observed in MscS/MscM and MscS/MscL double nulls, although the latter is complicated by the fact that the MscL null is associated with an inserted chloramphenicol resistance gene that should give the strain an inherent partial resistance. The mean CFU/mL for aerosolization time for 0, 5, 10, 20, and 30 min per strain (within strains) was not significant for 30 out of 42 hypothesis tests performed. Based on these experiments, strain 2 (Frag1-parent strain for mutant knockouts) and strain 4 (deletion of MscS channel) culturability decreased when aerosolization time increased. For strain 2, increased aerosolization stress for 5 and 20 min resulted in less growth in TSA media compared to no-stress conditions and a recovery in growth at 20 and 30 min. The culturability of strain 7 (deletion of MscL and MscS channels) remained the highest for all the different 0, 5, 10, 20, and 30 min treatments for each antibiotic amended media with mean values of 1.68 × 10^9^, 2.03 × 10^9^, 1.73 × 10^9^, 3.34 × 10^9^, and 2.7 × 10^9^ CFU/mL, respectively. The culturability counts of the collected aerosolized *E. coli* strains compared between strains are reported below, indicating which tests are statistically significant. The *p*-values indicating statistical significance at 95% confidence are summarized below. In summary, the data show that channel expression influences the sensitivity and resistance observed relative to aerosolization stress.

### 3.4. Antibiotic Resistance

**Figure 4:**
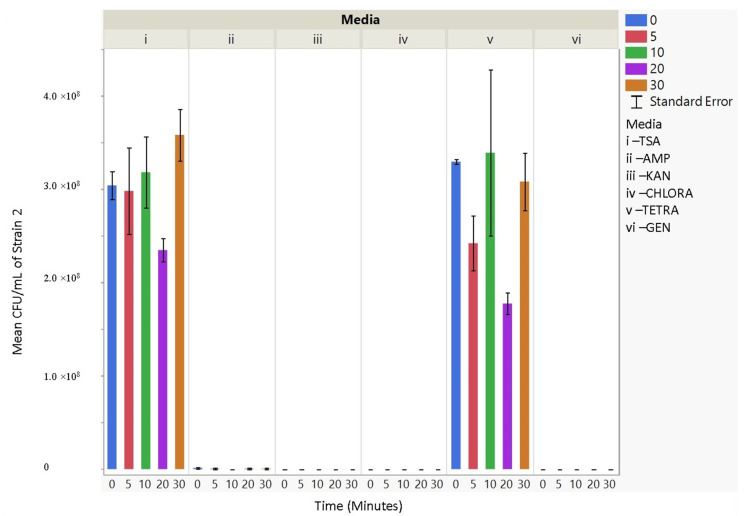
The mean CFU/mL values (left *Y*-axis) are shown for strain 2, Frag1, at 0, 5, 10, 20, and 30 min (bottom *X*-axis) per media (i, ii, iii, iv, v, and vi) (top *X*-axis).

**Figure 5:**
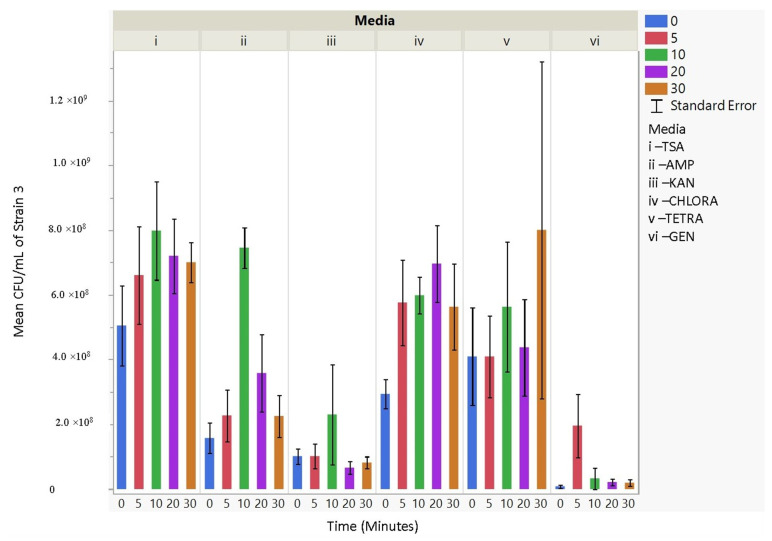
The mean CFU/mL values (left *Y*-axis) are shown for strain 3, MJF367, at 0, 5, 10, 20, and 30 min (bottom *X*-axis) per media (i, ii, iii, iv, v, and vi) (top *X*-axis).

**Figure 6:**
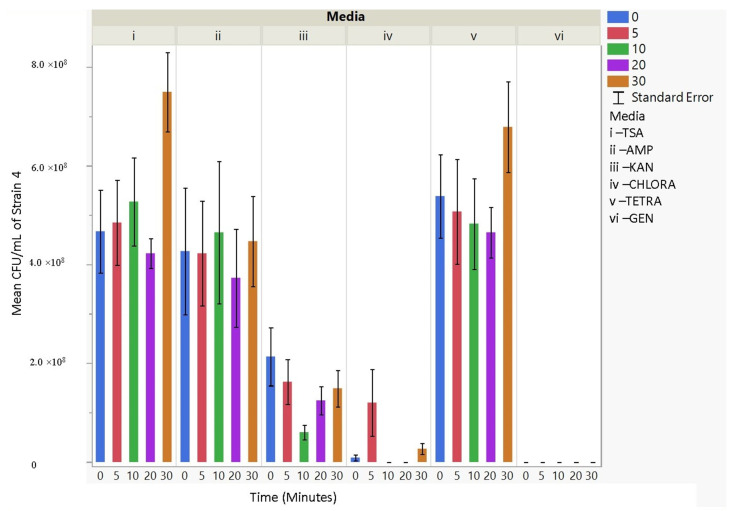
The mean CFU/mL values (left *Y*-axis) are shown for strain 4, MJF451, at 0, 5, 10, 20, and 30 min (bottom *X*-axis) per media (i, ii, iii, iv, v, and vi) (top *X*-axis).

**Figure 7:**
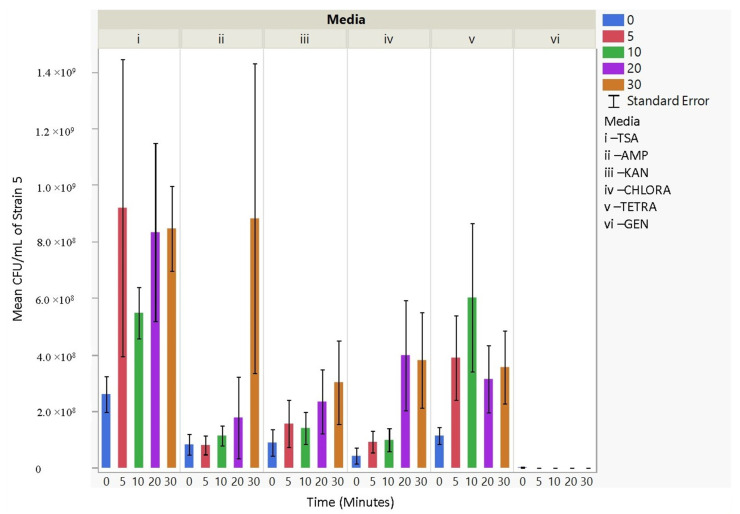
The mean CFU/mL values (left *Y*-axis) are shown for strain 5, MJF465, at 0, 5, 10, 20, and 30 min (bottom *X*-axis) per media (i, ii, iii, iv, v, and vi) (top *X*-axis).

**Figure 8:**
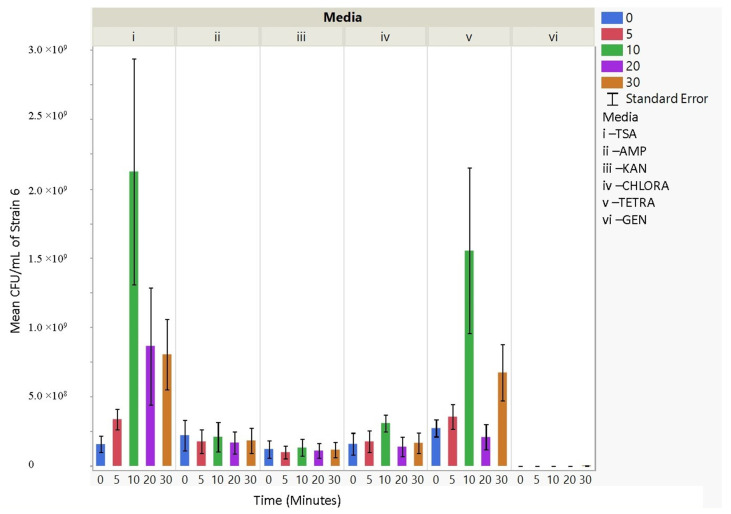
The mean CFU/mL values (left *Y*-axis) are shown for strain 6, MJF641, at 0, 5, 10, 20, and 30 min (bottom *X*-axis) per media (i, ii, iii, iv, v, and vi) (top *X*-axis).

**Figure 9:**
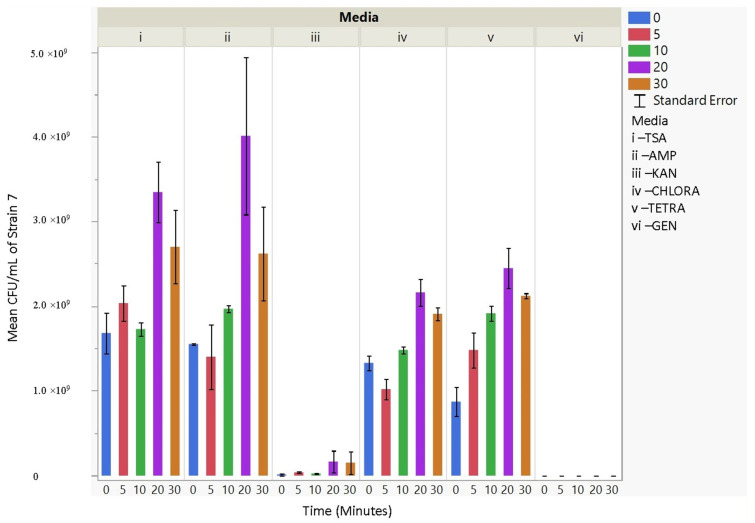
The mean CFU/mL values (left *Y*-axis) are shown for strain 7, MJF455, at 0, 5, 10, 20, and 30 min (bottom *X*-axis) per media (i, ii, iii, iv, v, and vi) (top *X*-axis).

The culturability (colony-forming units, CFU/mL) of each nebulized *E. coli* strain for different periods of time was examined for each antibiotic media (Figure 3, Figure 4, Figure 5, Figure 6, Figure 7, Figure 8 and Figure 9). The resistant responses of *E. coli* varied significantly in 21 out of 30 tests, indicating a difference in CFU/mL per antibiotic at 0, 5, 10, 20, or 30 min. Repeated susceptibility was seen as a response to kanamycin and gentamicin antibiotics for all *E. coli* strains. The parent strain (Frag1-strain 2) for the *E. coli* knockout strains exhibited susceptibility to ampicillin, kanamycin, chloramphenicol, and gentamicin (Figure 4). For Frag1, increased aerosolization stress for 5 and 20 min resulted in less growth in tetracycline compared to no-stress conditions and a recovery in growth at 20 and 30 min. These observations correspond to recent research in which it was highlighted that “Aerosolization triggers immediate antibiotic resistance in bacteria” in *E. coli* where recovery periods influence antibiotic uptake depending on the stress exposure [36]. Strain 3 (deletion of MscL channel) showed higher resistance for ampicillin and chloramphenicol (Figure 5), while Strain 4 (deletion of MscS channel) showed an increase in susceptibility to kanamycin and tetracycline as aerosolization stress increased (Figure 6). An increase in antimicrobial resistance was observed in strains 5 and 6 for tetracycline (Figure 7 and Figure 8) and in strains 1, 3, and 7 for ampicillin, chloramphenicol, and tetracycline (Figure 3, Figure 5 and Figure 9). Figure 8 shows a rapid initial recovery in culturability after 10 min, similarly to the results of our previous study where the bacterial response to stress included an immediate and a later mechanism (Smith and King, 2022). The *p*-values indicating statistical significance at 95% confidence are summarized below (Table 2, Table 3 and Table 4).

### 3.5. Hypothesis Testing

Several hypothesis tests were conducted to determine if there is a significant difference in average CFU/mL values of the *E. coli* strains.

## 4. Discussion

The examples in which bacterial MS channels influence infection and antibiotic resistance, and the mechanisms by which they do so, are only now being elucidated [37]. MS channels can serve as pathways for antibiotics to enter the cytoplasm, or they may release stresses that would otherwise increase antibiotic susceptibility. Some of the mechanisms by which MscL influences antibiotic sensitivity have been studied only recently [25]. Changes in MS channel expression, when combined with changes in osmolarity [38], cell wall integrity via ampicillin treatment [39,40], or even freezing [41], have been shown to influence antimicrobial susceptibility. Thus, it may not be too surprising that changes in specific MS channel expression, when combined with aerosolization, also influence antibiotic resistance. The MscM, MscS, and MscL channels, which are all easily observed by patch clamp, appear to play key roles in this phenomenon. The observation that they can either increase sensitivity or resistance suggests that different channels and paralogues within *E. coli* can have different influences on antibiotic resistance. While it seems likely from previous studies that these influences are direct, we cannot rule out the possibility that compensatory mechanisms, including the regulation of other channel expressions within the cell, may contribute to the overall response of microorganisms.

The *E. coli* strains that were aerosolized demonstrated the capability to promptly recover antibiotic resistance and culturability despite increased exposure to stress. Cells from each *E. coli* strain experienced rapid environmental changes from 37 °C to aerosolization at 20 pounds per square inch (psi), and air transport at 12.5 L/min at 5, 10, 20, and 30 min. This rapid change in external pressure and velocity caused a higher stress response from the cells relative to the time of exposure to stress, as indicated by the average CFU/mL counts. Strain 1 at 5 and 20 min aerosolization displayed different behaviors in antibiotic susceptibility to ampicillin, while strain 3 at 10 min aerosolization displayed different behaviors in antibiotic susceptibility testing for chloramphenicol media. The phenotypic behavior can be related to prolonged stress, which triggers a metabolic response to regulate survival mechanisms in cells. Interestingly, when *E. coli* mutant knockout strains were nebulized for longer durations (20–30 min), resistance increased. However, it should be noted that any strain null for MscL should have resistance to chloramphenicol due to how the null strain was produced. Further molecular methods are required to clarify the internal factors that may affect the culturability and AMR of *E. coli.* This study hypothesized that increased *E. coli* mutant knockout genes of mechanosensitive channels would behave differently under prolonged aerosolization stress, as shown by the antimicrobial response. Further studies should focus on gene expression analysis using whole genome sequencing for changes in genes regulating the stress response and identifying metabolomics of *E. coli* strains under high-velocity conditions to clarify how cells regulate their stress response.

## 5. Conclusions

There are still questions to be addressed regarding the uncertainties of real-time bacterial defense mechanisms and their detection ability. There is an identifiable gap in the ambiguity of how bacteria change in their natural environment and in laboratory settings. This gap affects the development of genetic tests targeting mutations and statistical analyses of the results. The results of this study can help clarify how stable genetic mutations behave in a ventilated microbial genome and clarify their suitability as genetic markers. It is unclear how likely it is that the same genetic mutations will emerge independently in separate cultures; in this study, we evaluated whether different culture conditions can change the ratio of gene expression. An important observation to keep in mind is whether different culture conditions can alter the ratio of mutations significantly enough to provide a negative rather than a positive result. This study clarifies the significance of using mutations as genetic markers for analyzing sample and strain behavior. Specifically, we determined how mscL/S gene presence is related to other defense mechanisms displayed by AMR pathogens. The next step would be to target mechanosensitive channels as antimicrobial points of entry and observe how they are stressed. Future investigations will identify more potential triggers leading to mechanisms of resistance in cells as they are related to physical stresses by airflow parameters. The physiological role of bacterial mechanosensitive channels has been documented; they serve as emergency valves upon exposure of bacteria to hypo-osmotic environments [15]. Furthermore, evidence suggests that the expression of bacterial mechanosensitive channels may be related to cell wall remodeling during the stationary phase of cell growth [42]. This study clarifies whether environmental stressors on *E. coli* MG1655 mechanosensitive channels lead to gene expression that regulates the cell wall permeability of antimicrobials.

## Figures and Tables

**Table 1:** Bacterial strains of *Escherichia coli* utilized in this study.

Strain	Gene Deletion Annotation	Description
MG1655		Wild type
2. Frag1		Parent strain for mutant knockouts
3. MJF367	Frag1, *ΔmscL::Cm*	Deletion of MscL channel
4. MJF451	Frag1, *ΔyggB*	Deletion of MscS channel
5. MJF465	Frag1, *ΔmscL::Cm, ΔyggB, ΔkefA::ka*	Deletion of MscL, MscS, and MscK/KefA channels
6. MJF641	Frag1, (Δ7) *mscS-mscK-ybdG-ybiO-yjeP-ynaI-mscL*	Deletion of all 7 MS channels
7. MJF455	Frag1, *ΔmscL::Cm, ΔyggB*	Deletion of MscL and MscS channel

**Table 2:** Hypothesis testing mean µ CFU/mL between strains.

0 min CFU/mL vs. strain per media	
Test 1: TSA *p*-value < 0.0001	Test 4: CHLORA *p*-value = 0.0002
Test 2: KAN *p*-value < 0.0001	Test 5: TETRA *p*-value < 0.0001
Test 3: AMP *p*-value < 0.0001	Test 6: GEN *p*-value = 0.1751
5 min CFU/mL vs. strain per media	
Test 7: TSA *p*-value = 0.0775	Test 10: CHLORA *p*-value < 0.0001
Test 8: KAN *p*-value = 0.6497	Test 11: TETRA *p*-value < 0.0001
Test 9: AMP *p*-value < 0.0001	Test 12: GEN *p*-value = 0.0901
10-min CFU/mL vs. strain per media	
Test 13: TSA *p*-value = 0.0491	Test 16: CHLORA *p*-value < 0.0001
Test 14 KAN *p*-value = 0.4625	Test 17: TETRA *p*-value = 0.0466
Test 15: AMP *p*-value < 0.0001	Test 18: GEN Media = 0.6090
20 min CFU/mL vs. strain per media	
Test 19: TSA *p*-value < 0.001	Test 22: CHLORA Media < 0.0001
Test 20: KAN *p*-value = 0.3189	Test 23: TETRA Media < 0.0001
Test 21: AMP *p*-value < 0.0001	Test 24: GEN Media = 0.0558
30 min CFU/mL vs. strain per media	
Test 25: TSA *p*-value < 0.0001	Test 28: CHLORA *p*-value < 0.0001
Test 26: KAN *p*-value = 0.2004	Test 29: TETRA *p*-value = 0.0038
Test 27: AMP *p*-value = 0.0002	Test 30: GEN *p*-value = 0.0336

Ho: µ_strain 1_ = µ_strain 2_ = µ_strain 3_ = µ_strain 4_ = µ_strain 5_ = µ_strain 6_ = µ_stain 7_; Ha: The mean µ (CFU/mL) is not equal; A *p*-value less than 0.05 is statistically significant. This indicates strong evidence against the null hypothesis, i.e., the means between strains are different.

**Table 3:** Hypothesis testing mean µ within strains.

Strain 1: MG1655 at 0, 5, 10, 20, and 30 min	
Test 31: TSA *p*-value = 0.3314	Test 34: CHLORA *p*-value = 0.3562
Test 32: KAN *p*-value = 0.0390	Test 35: TETRA *p*-value = 0.1396
Test 33: AMP *p*-value = 0.2897	Test 36: GEN *p*-value = 0.4220
Strain 2: Frag at 1 0, 5, 10, 20, and 30 min	
Test 37: TSA *p*-value = 0.1575	Test 40: CHLORA µ_min 0_ = µ_min 5_ = µ_min 10_ = µ_min 20_ = µ_min 30_ = 0
Test 38: KAN µ_min 0_ = µ_min 5_ = µ_min 10_ = µ_min 20_ = µ_min 30_ = 0	Test 41: TETRA *p*-value = 0.0331
Test 39: AMP *p*-value = 0.6554	Test 42: GEN p µ_min 0_ = µ_min 5_ = µ_min 10_ = µ_min 20_ = µ_min 30_ = 0
Strain 3: MJF367 at 0, 5, 10, 20, and 30 min	
Test 43: TSA *p*-value = 0.5528	Test 46: CHLORA *p*-value = 0.1033
Test 44: KAN *p*-value < 0.0001	Test 47: TETRA *p*-value = 0.7711
Test 45: AMP *p*-value = 0.4813	Test 48: GEN Media *p*-value = 0.0357
Strain 4: MJF451 at 0, 5, 10, 20, and 30 min	
Test 49: TSA *p*-value = 0.0405	Test 52: CHLORA *p*-value = 0.0408
Test 50: KAN *p*-value = 0.1156	Test 53: TETRA *p*-value = 0.4408
Test 51: AMP *p*-value = 0.9868	Test 54: GEN µ_min 0_ = µ_min 5_ = µ_min 10_ = µ_min 20_ = µ_min 30_ = 0
Strain 5: MJF465 at 0, 5, 10, 20, and 30 min	
Test 55: TSA *p*-value = 0.4457	Test 58: CHLORA *p*-value = 0.0976
Test 56: KAN *p*-value = 0.5632	Test 59: TETRA *p*-value = 0.3060
Test 57: AMP *p*-value = 0.1511	Test 60: GEN µ_min 0_ = µ_min 5_ = µ_min 10_ = µ_min 20_ = µ_min 30_ = 0
Strain 6: MJF641 at 0, 5, 10, 20, and 30 min	
Test 61: TSA *p*-value = 0.0222	Test 64: CHLORA *p*-value = 0.5003
Test 62: KAN *p*-value = 0.9937	Test 65: TETRA *p*-value = 0.0112
Test 63: AMP *p*-value = 0.9951	Test 66: GEN µ_min 0_ = µ_min 5_ = µ_min 10_ = µ_min 20_ = µ_min 30_ = 0
Stain 7: MJF455 at 0, 5, 10, 20, and 30 min	
Test 67: TSA *p*-value = 0.0101	Test 70: CHLORA *p*-value = 0.0001
Test 68: KAN *p*-value = 0.5573	Test 71: TETRA *p*-value = 0.0004
Test 69: AMP *p*-value = 0.0487	Test 72: GEN µ_min 0_ = µ_min 5_ = µ_min 10_ = µ_min 20_ = µ_min 30_ = 0

Ho: µ_min 0_ = µ_min 5_ = µ_min 10_ = µ_min 20_ = µ_min 30_; Ha: The mean µ (CFU/mL) is not equal; A *p*-value less than 0.05 is statistically significant. This indicates strong evidence against the null hypothesis, i.e., the means between strains are different.

**Table 4:** Summary of significance.

Difference between mean CFU/mL of strains	0 min CFU/mL vs. strains 1, 2, 3, 4, 5, 6, 7 TSA, KAN, AMP, CHLORA, TETRA 5 min CFU/mL vs. strains 1, 2, 3, 4, 5, 6, 7 AMP, CHLORA, TETRA 10 min CFU/mL vs. strains 1, 2, 3, 4, 5, 6, 7 TSA, AMP, CHLORA, TETRA 20 min CFU/mL vs. strains 1, 2, 3, 4, 5, 6, 7 TSA, AMP, CHLORA, TETRA 30 min CFU/mL vs. strains 1, 2, 3, 4, 5, 6, 7 TSA, AMP, CHLORA, TETRA, GEN
Difference within mean CFU/mL of strains	Strain 1: MG1655 at 0, 5, 10, 20, and 30 min KAN Strain 2: Frag 1 at 0, 5, 10, 20, and 30 min TETRA Strain 3: MJF367 at 0, 5, 10, 20, and 30 min KAN, GEN Strain 4: MJF451 at 0, 5, 10, 20, and 30 min TSA, CHLORA Strain 6: MJF641 at 0, 5, 10, 20, and 30 min TSA, TETRA Stain 7: MJF455 at 0, 5, 10, 20, and 30 min TSA, AMP, CHLORA, TETRA

## Data Availability

Research data will be shared by the Corresponding Author upon request.
